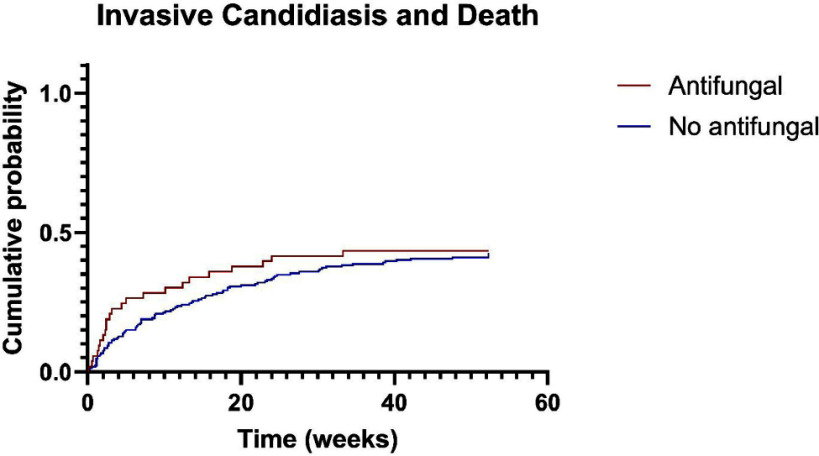# Antifungal use following Candida Growth in Bile Cultures Collected during Endoscopic Retrograde Cholangiopancreatography

**DOI:** 10.1017/ash.2025.256

**Published:** 2025-09-24

**Authors:** Kevin Smith, Grace Charpentier, Rachel Susler, Theresa Emeli, Nicolas Barros

**Affiliations:** 1 Indiana University School of Medicine; 2 Indiana University School of Medicine; 3 Indiana University School of Medicine; 4 Indiana University

## Abstract

**Background:** Candida species are increasingly causing infections and are considered high priority fungal pathogens. Despite this, published data describing the clinical importance of Candida growth in the bile tract is limited to case reports and small cohorts. Our goal is to characterize treatment outcomes of patients who had Candida spp isolated from bile cultures obtained via Endoscopic Retrograde Cholangiopancreatography (ERCP) to determine the value of antifungal use in these cases. **Methods:** We performed a single-center retrospective cohort study of patients with bile cultures positive for Candida spp collected during ERCPs from January 2010 to December 2023. Patients were identified by cross-matching databases of patients who underwent ERCP and patients with Candida-positive bile cultures. The treatment cohort included patients who received antifungals within seven days of the first ERCP with Candida growth in bile cultures (principal ERCP) compared to a control cohort who did not. Patients with candidemia or deep-seated candida infection prior to the principal ERCP or insufficient chart data were excluded. The primary outcome was a composite of death and/or development of invasive candida infection within one year of the principal ERCP date. Kaplan Meier plots and log-rank tests were used to analyze the primary outcome. **Results:** A total of 266 patients were included out of 285 with 8 being excluded for insufficient records and 11 being excluded for invasive candidiasis within one year prior. The included patient population was 60.2% male, 79.7% white, 7.9% black, and 12.4% other/unknown race and had a mean age of 63.6 +/- 15.8 years. The most common species of Candida identified were C albicans (65.9%), C glabrata (17.4%), and C tropicalis (7.2%) with 27 patients (9.2%) having 2 isolates in their culture. There were 52 patients (19.5%) who received antifungals—46 fluconazole and 6 micafungin. At one year the primary endpoint occurred in 23 out of 53 patients (43.3%) in the antifungal group and 93 out of 213 patients (43.6%) in the control group. The primary outcome was plotted on a Kaplan Meier curve. The hazard ratio was 1.14 (0.71 to 1.85, 95% CI; p=0.574) which did not reach statistical significance. Additionally, antifungal initiation had no statistically significant impact on rehospitalization rates (p=0.602), relapse of bacterial cholangitis (p=0.230), or recurrent Candida-positive bile cultures (p=0.441) within one year. **Conclusions:** This retrospective study of the use of antifungals in patients with Candida growing from bile cultures following ERCP found no benefit in starting antifungal treatment.

**Figure:**